# Introducing biomarker panel in esophageal, gastric, and colon cancers; a proteomic approach 

**Published:** 2015

**Authors:** Mona Zamanian–Azodi, Mostafa Rezaei–Tavirani, Hadi Hasanzadeh, Sara Rahmati Rad, Sona Dalilan

**Affiliations:** 1 *Proteomics Research Center, Faculty of Paramedical Sciences, Shahid Beheshti University of Medical Sciences, Tehran, Iran*; 2 *Department of Medical Physics, Semnan University of Medical Sciences, Semnan, Iran*; 3 *Department of Cell and Molecular Biology, Faculty of Science, University of Tehran, Tehran, Iran*; 4 *Department of Science and Researches, Islamic Republic University, Tehran, Iran*

**Keywords:** Biomarker panel, Esophagus cancer, Gastric cancer, Colon cancer, Proteomics

## Abstract

Cancer research is an attractive field in molecular biology and medicine. By applying large-scale tools such as advanced genomics and proteomics, cancer diagnosis and treatment have been improved greatly. Cancers of esophagus, gastric, and colon accounted for major health problem globally. Biomarker panel could bring out the accuracy for cancer evaluation tests as it can suggest a group of candidate molecules specified to particular malignancy in a way that distinguishing malignant tumors from benign, differentiating from other diseases, and identifying each stages with high specificity and sensitivity. In this review, a systematic search of unique protein markers reported by several proteomic literatures are classified in their specific cancer type group as novel panels for feasible accurate malignancy diagnosis and treatment. About thousands of introduced proteins were studied; however, a small number of them belonged to a specific kind of malignancy. In conclusion, despite the fact that combinatorial biomarkers appear to be hopeful, more evaluation of them is crucial to achieve the suitable biomarker panel for clinical application. This effort needs more investigations and researches for finding a specific and sensitive panel.

## Introduction

Cancer is the third foremost cause of death worldwide (1,2). There is no proper early detection and treatment methods available relative to various cancers (3). Gastrointestinal cancers are among the most rampant malignancies and are fatal if remained uncured (4). Common treatments are chemotherapy, radiotherapy, and surgery (5, 6). In 1847, the study of cancer biomarker proteins started by Henry Bence-Jones (7). Mutated proteins as the ultimate products of gene expression, not only related to tumors but also to tumorigenesis (8). These elements can play an integral role in many approaches in cancer studies such as diagnosis, detection, patient monitoring, treatment, and tumor classification (-); pricey, invasive cancer diagnostic tests can be substituted by applying reasonable explicit biomarker test (13). Biomarker test started to demonstrate its novel feature in the detection and management of patients with different malignancies (14,15). High-throughput techniques can be valuable in this field (16). Proteomics as a large-scale protein analyzers is one of the promising tools, enabling a researcher to evaluate malignancies at the molecular stage (17). This method is a new developing ultrasensitive detector while other protein assay tools can just detect ranges about the sub pg mL^−1^ level (18). Furthermore, in the field of cancer proteomics study, comprehensive analysis of protein interactions, expression, and functions for identifying biomarkers (19), pathways, primary tumors and their metastases relationship and tumor heterogeneity, disease classification, understanding of disease development, and assessment of treatment responses are the main concerns (20, 21). 

Sensitivity and specificity in determining biomarkers are crucial due to clinical cancer detection, surveillance after treatment, and therapy selection (22). Protein marker panel could be useful in distinguishing malignant from benign, differentiate from other diseases, and identifying each stages with high specificity and sensitivity. In other words, this preliminary data, if validated in larger clinical studies, could be developed into a serum protein test with less falsely positive diagnoses in which decreases the number of unnecessary biopsies and identifies individuals who need treatment before symptoms begin (23, 24). Since early detection in gastrointestinal cancers is crucial for therapeutic management (25), in this review, selective cancer protein markers and their properties (-) in different common gastrointestinal duct cancers including esophagus, gastric, and colon neoplasm (29, 30) are chosen and discussed for achieving to a proper protein panel for clinical purposes. 

**Esophageal Cancer**

Esophageal cancer as one of the most common malignancies in the world has a wide area broaden from the southern border of the Caspian Sea in Iran across central Asia to China (31). It is accounted for the sixth cause of death in the world with the more than 50 percent metastatic condition at the time of detection (32). The most common type is esophageal squamous cell carcinoma (ESCC) with increasing rate in Western countries (33), due to increase rate of obesity and gastroesophageal reflux disease (GERD) (34). Another type is adenocarcinoma (EAC), which is lethal and occur either within Barrett’s epithelium or in the gastric cardiac mucosa of the distal esophagus (35). Regularly, symptoms start with dysphagia, weight loss, chest pain, pressure or burning, and coughing (-). Many risk factors are accompanied with this malignancy such as diet, smoking, Gastroesophageal reflux disease (GERD), and *human papilloma virus *(HPV) (-)*.* Methods for detection of esophageal cancer are Esophagoscopy, endoscopic optical, positron emission tomographyraman spectroscopy, immunohistochemistry, and biopsy. The last method (biopsy) is a histological examination for confirming physical and imaging tests (-). General treatment options are photodynamic therapy, chemotherapy, radiotherapy, chemoradiotherapy, and surgery (Esophagectomy), which is the most efficient choice (46). A number of protein markers have been identified for esophageal detection are listed as below (table1). 

**Gastric Cancer**

Gastric cancer (GC) as the fifth most common malignancies in the world (59, 60) is the second primary cancer related lethal causing (-); in addition, about 800,000 cancer-related deaths are caused by gastric cancer each year worldwide announced by the World Health Organization (64). Due to late diagnosis, limited therapy options for most patients, the rate of 5-year survival is less than 20%. The most common type of stomach cancer is adenocarcinomas, which is divided into diffuse (undifferentiated) and intestinal (well-differentiated) and is about 90% of all; the rest are gastric lymphoma, gastrointestinal stromal tumors, and neuroendocrine tumors with about 3-5% of all malignant tumors of the stomach (65, 66) with high incident in  Asian countries (67). There are two types of gastric adenocarcinoma based upon anatomical location: cardia or proximal, and distal, non-cardia adenocarcinomas (68). The primarily carcinogen is known as *Helicobacter pylori* infection, which is considers as 60 to 70% of gastric cancer occurrence in the world. Studies showed that eradication of *H.pylori* has been successful in reducing gastric cancer risk (3). The prognosis is still not sufficient even though its incident has been declined in recent years in the United States. Regular methods for early detection and screening stomach cancer are ultrasonography, endoscopic sonography, double contrast radiologic method, computed tomography (CT), or magnetic resonance imaging (-). Some protein markers related to gastric cancer are tabulated in table 2. 

**Table1:** Biomarker panel related to esophagus cancer, the MeSH terms (NBCI Databases) of these markers are mentioned.

Disease	Protein name	Category	Function	Condition	Application
Esophageal squamous cell carcinoma and Adenocarcinomas (47)	MAP3K3 protein (48), also known as: MEKK3 protein, human mitogen-activated protein kinase kinase kinase 3, human MAPKKK3 protein, human	Mitogen-activated protein (49)	Regulators of nuclear factor kappa B (NF-κB) (48)	Up-regulated (47)	Early detection (48)
Esophageal squamous cell carcinoma (50)	(Jarid1b) (51), also known as: Jumonji, AT rich interactive domain 1B protein, human RBP2-like protein, human PLU-1 protein, human PLU1 protein, human RBBP2H1A protein, human JARID1B protein, human lysine (K)-specific demethylase 5B, human	Jumonji/Arid1b family (52)	Promotes human esophagealcancer cell growth (53)	Up-regulated(54)	Diagnosis(55)
Esophageal squamous cell carcinoma (56)	UNC-51 like kinase 1 also known as ATG1 protein (56)	Protein kinase superfamily(57)	Autophagosmoe formation (56)	Up-regulated (56)	Diagnosis (58)

**Colon Cancer**

Colorectal cancer is the second cause of cancer death with 30% inheritance base (89); in fact, half of all patients diagnosed with this invasive cancer would ultimately die (90, 91). Therefore, the prognosis is highly linked to early diagnosis; it leads to a five-year survival post-operation of over 80% while in the advanced conditions the five-year survival decreases to 40% (92). 

**Table 2:** The summarized protein markers specific to gastric cancer, the MeSH terms (NBCI Databases) of these markers are mentioned.

Disease	Protein name	Category	Function	Condition	Application
Gastric carcinoma (72), Adenomas(73)	Gastrokines :Gastrokine 1 (GKN1) (74, 75) also known Antrum mucosal protein (AMP)-18 (76) GKN2 (77), GKN3(78)	Family of stomach-specific proteins (79)	Growth inhibition (75), Mucosal protection (72)	Down-regulated (80)	Early detection(81)
Gastric carcinoma(82)	Pepsinogen C (PGC)(83)also known as Gastricsin	Belongs to the peptidase family A1	Aspartic proteinase(84)	Low expression(85)	Early detection(83)
Gastric carcinoma(86)	IPO-38 antigen(87, 88), also known as N-L116 antigen	Belongs to H2B histone(87)	Proliferatingmarker(87)	Up-regulated(88)	Early detection(87)

**Table 3:** The putative tumor markers related to colon cancer.

Disease	Protein name	Category	Function	Condition	Application
Colorectalcarcinoma	Proteasome subunit beta type 7 (PSB7) (104)	Cytoplasmic and nuclear protein(105)	Integral to cellular proteolytic degradation capability(106)	Up-regulated(105)	Early detection(105)
Colorectal adenocarcinoma(107)	Colon cancer-specificantigens : (CCSA)-2(108) (CCSA)-3(109) (CCSA)-4(110) (CCSA)-5 (111)	Nuclear matrix proteins (NMPs) (102)	Structural role in nuclear(109)	Up-regulated(108)	Early detection(112)

Adenocarcinoma is the most frequent type of colon cancer (93), the rest includes gastrointestinal carcinoid tumors, gastrointestinal stromal tumors, primary colorectal lymphoma, leiomyosarcoma, melanoma and squamous cell carcinoma (-). Rectal bleeding, diarrhea, weight loss, abdominal pain, and anemia are considered to be the common symptoms of this malignancy (98). Risk factors that are accompanied with this disease are smoking and red meat consumption. On the other hand, the risk of colon cancer can be reduced by Aspirin (99, 100), consuming vegetables, grains, and fruit (101). Mainly colorectal cancer detection is divided into two category: 1) guaiac fecal occult blood testing (gFOBT), fecal immunochemical test (FIT) and testing stool for exfoliated DNA (sDNA) that is, stool test; and 2) Detecting lesions by methods including flexible sigmoidoscopy (FSIG), colonoscopy (CSPY), double-contrast barium enema (DCBE), computed tomography colonography (CTC), and colonoscopy (102). In addition, colonoscopy is an aggressive approach with approximately 90% effectiveness and not being able to detect all colonic polyps(103). These applications by being costly and invasive, causing possible bleeding or infection, bowel irritating, and possessing low specificity render to search for safer techniques providing minimum invasion, high performance, high degree of sensitivity and specificity, and inexpensive. In table 3, some of the putative protein markers of colorectal cancer are included (table3).

## Discussion

Biomarkers are efficient diagnostic agents for detection and even early detection of diseases, especially malignant diseases (9,-). Biomarkers are diverse biochemical components such as DNA sequence, RNAs, proteins, and metabolites (116). The final goal of biomarker application is to introduce a sensitive and specific indicator for a certain kind of disease. For many kinds of diseases, this strategy is offered. For instance, prostate specific antigen (PSA) is a regular diagnostic biomarker for prostate cancer (117). Unfortunately, there is no specific biomarker for many kinds of malignant diseases (118,119). Therefore, intensive efforts for introducing a combination of various biomarkers (biomarker panel) are in progress (120,121). Biomarker panel study has been started to gain great attention in recent years (122). A single biomarker cannot be examined as accurate diagnosis tool due to cancer diversity, since several of them are reported in other diseases. Thus, the specificity and sensitivity of the evaluation can decrease. In a new approach combination of markers are applied to provide the efficient accuracy while a single of them is inadequate. These selected tumor markers should bring out less commonness between each cancers and other diseases especially cancer of same histological origin. In this study, these markers were selected based on most reported as a preferentially expressed marker in definite cancer during recent years. Each of these markers is chosen as independent marker, but it is necessary to combine and present them as a set of markers. Since these biomarkers may be related to other cancers, an accurate clinical examination can be a useful tool in distinguishing between different types of malignancies. Furthermore, the selected protein markers for the panel study are those that are observed in neoplasm with less histological origin. For example, some of these markers are seen in both esophageal and gastric cancers, so they were omitted for this research. Several proteins reported as candidate biomarkers for these cancers; nevertheless, not all represent the accurate diagnosis factors since they are common with other cancers. For clinical applications, more efforts should be done to managing these findings. In table 1, three markers related to esophagus cancer were achieved among them, (MAP3K3/MEKK3) over-expression plays a significant role in tumorigenesis (123). 

This biomarker is also reported in other malignancies such as breast cancer and hepatocellular carcinoma (HCC) (124,125). The other marker, Jumonji/Arid1b (Jarid1b) protein modulates cell growth and its function is highly increased in esophageal, breast, melanoma, and prostate cancer (53, -). Protein marker, UNC-51 like kinase 1 can be also a specific diagnosis marker for esophageal neoplasm              (56). As it is mentioned in table 2, gastrokine family has an explicit role in gastric malignancy (-). Among these GKNs, gastrokin 3 is recently discovered (131). GKN1 also called antrum mucosal protein (AMP)-18     (76) is one of the most putative biomarkers (132). Its low expression can be concluded in onset of malignancy. Moreover, while in its presence in *Helicobacter pylori-*negative patients reported normal, it is decreased in *Helicobacter pylori*-positive patients (133). In addition, pepsinogen C plays a prominent role in gastric cancer (134). This marker is also reported to be an important marker in different gastric diseases. Its expression in superficial gastritis is completely lost while in other diseases such as gastric ulcer or erosion was reduced to 80%. On the other hand, this reduction is about 11% in gastric cancer (85). 

Additionally, it is a known marker for breast cancer, and prostate cancer (135). IPO-38 that is expressed frequently through most stages of the cell cycle except during mitosis is another novel gastric tumor marker. It is also known as N-L116 antigen in many databases (136), and assigned for ameloblastomas malignancy (137). In addition, lots of highly reported gastric biomarkers such asReg IV (138, 139), serum amyloid A (SAA) (140, 141), Adipocytokine (142, 143),  sphingosine kinase 1 (SK1) (144, 145), and SM22 (146, 147) were observed during this review, but all of them were correlated with colon cancer as well. Thereby, in this study they are not considered as definite tumor marker due to having the same histological bases. As it is shown in table3, colon cancer biomarkers are introduced as specific markers including PSB7, colon-cancer specific antigen family. Colon cancer specific antigens including (CCSA)-2 (108), (CCSA)-3 (109),(CCSA)-4 (110), and (CCSA)-5        (111) are assigned with high sensitivity and specificity that are detectable in serum with high quantity. These potential markers are reported by many proteomic analyses (108, 109). Among all of these evaluated biomarkers, solely colon cancer biomarkers showed definite specificity comparing with esophageal cancer and gastric cancer. Most discovered tumor markers are not appropriate as standard clinical methods (148); a selected group of them could be useful as a feasible tool for clinical approaches. As it is reported in numerous researches, there are hundreds of nonspecific biomarkers for digestive system cancers. However, a small number of these proteins are merely observed in the nature of these cancers. More follow-up evaluations are required to lessen limitations in selective biomarker applications in clinical approaches. These verifications can be achieved via clinical examinations researches. Large-scale studies such as advanced proteomics can introduce new relevant biomarkers for achieving efficient panel (149). It can be concluded that non-invasive methods such as proteomics provides a great number of biomarkers (-) that can be managed by clinical approaches for efficient prognosis and diagnostic purposes in a format of biomarker panel. It seems that biomarker panels play an important role in cancer management especially in diagnostic procedures in near future.

## Conclusion

Nemours protein biomarkers are introduced for esophageal, gastric and colon cancers. It seems that each of them has no adequate potential for clinical application due to insufficient specificity and sensitivity. This valuable source of biomarkers can be considered for formation of biomarker panels, which are useful and precious tools in cancer management. In general, biomarker analysis and clinical examination are two important activities that can lead to introducing novel biomarker panel for accurate prognosis, diagnosis, and patients follow up.
